# Covid-19 Vaccine Hesitancy and Under-Vaccination among Marginalized Populations in the United States and Canada: A Scoping Review

**DOI:** 10.1007/s40615-023-01882-1

**Published:** 2023-12-20

**Authors:** Peter A. Newman, Duy A. Dinh, Thabani Nyoni, Kate Allan, Sophia Fantus, Charmaine C. Williams, Suchon Tepjan, Luke Reid, Adrian Guta

**Affiliations:** 1 Factor-Inwentash Faculty of Social Work, University of Toronto, Toronto, ON Canada; 2 Faculty of Health Sciences, Queen’s University, Kingston, ON Canada; 3 School of Social Work, University of Texas at Arlington, Arlington, TX USA; 4 VOICES-Thailand Foundation, Chiang Mai, Thailand; 5 School of Social Work, University of Windsor, Windsor, ON Canada

**Keywords:** Vaccine hesitancy, Vaccination, Covid-19 vaccines, Structural racism, Institutional mistrust, Access to care, Ethnic and racial minorities

## Abstract

**Background:**

Amid persistent disparities in Covid-19 vaccination and burgeoning research on vaccine hesitancy (VH), we conducted a scoping review to identify multilevel determinants of Covid-19 VH and under-vaccination among marginalized populations in the U.S. and Canada.

**Methods:**

Using the scoping review methodology developed by the Joanna Briggs Institute, we designed a search string and explored 7 databases to identify peer-reviewed articles published from January 1, 2020–October 25, 2022. We combine frequency analysis and narrative synthesis to describe factors influencing Covid-19 VH and under-vaccination among marginalized populations.

**Results:**

The search captured 11,374 non-duplicated records, scoped to 103 peer-reviewed articles. Among 14 marginalized populations identified, African American/Black, Latinx, LGBTQ+, American Indian/Indigenous, people with disabilities, and justice-involved people were the predominant focus. Thirty-two factors emerged as influencing Covid-19 VH, with structural racism/stigma and institutional mistrust (structural)(n = 71) most prevalent, followed by vaccine safety (vaccine-specific)(n = 62), side effects (vaccine-specific)(n = 50), trust in individual healthcare provider (social/community)(n = 38), and perceived risk of infection (individual)(n = 33). Structural factors predominated across populations, including structural racism/stigma and institutional mistrust, barriers to Covid-19 vaccine access due to limited supply/availability, distance/lack of transportation, no/low paid sick days, low internet/digital technology access, and lack of culturally- and linguistically-appropriate information.

**Discussion:**

We identified multilevel and complex drivers of Covid-19 under-vaccination among marginalized populations. Distinguishing vaccine-specific, individual, and social/community factors that may fuel decisional ambivalence, more appropriately defined as VH, from structural racism/structural stigma and systemic/institutional barriers to vaccination access may better support evidence-informed interventions to promote equity in access to vaccines and informed decision-making among marginalized populations.

**Supplementary Information:**

The online version contains supplementary material available at 10.1007/s40615-023-01882-1.

## Introduction

Vaccination is an essential component of public health strategies to control the Covid-19 pandemic. Vaccination reduced Covid-19-related morbidity and mortality in the United States (U.S.) [1], and globally—with an estimated 63% (14.4–19.8 million) reduction in deaths in the first year of Covid-19 vaccination alone [2]. Vaccine booster doses [3], and bivalent vaccine boosters compared with earlier monovalent vaccination [4], have further reduced Covid-19-associated hospitalization and deaths. Yet, despite the tremendous successes of Covid-19 vaccination, pervasive challenges ascribed to vaccine hesitancy (VH), and broader Covid-19 under-vaccination, threaten the effectiveness of vaccines in controlling Covid-19 and other vaccine-preventable diseases.

Disparities in Covid-19 morbidity and mortality are well documented among marginalized populations in the U.S. [5–8] and Canada [9–12]. Marginalization refers to systemic processes driven by ideologies such as racism, sexism, heterosexism, cissexism, ableism, xenophobia, and other forms of structural stigma that exclude certain populations or relegate them to the periphery of political and socioeconomic resources [13–15]. The structural and social inequities produced by marginalization contribute to adverse social determinants of health (SDOH)—such as residential segregation and housing insecurity, employment precarity, lack of access to affordable healthcare, lack of accessibility accommodations—which increase vulnerability to poor health outcomes [13, 16]. Adverse SDOH increase vulnerability to Covid-19 among marginalized populations via multiple pathways: disproportionate prevalence of underlying health conditions (i.e., diabetes, heart disease, chronic obstructive pulmonary disease, etc.) that are associated with worse Covid-19 outcomes; lower levels of access to healthcare (i.e., due to residential segregation) and health insurance coverage (i.e., due to employment precarity/unemployment); and by impeding the ability to practice public health measures (e.g., physical distancing, work-from-home) to reduce viral transmission [7, 17–22].

Heightened vulnerability to Covid-19 indicates an increased need for vaccination; however, the same adverse SDOH that drive disparities in Covid-19 outcomes may contribute to *lower* levels of vaccination [22, 23]. In fact, lower rates of Covid-19 vaccination have been reported among racialized and other marginalized populations in the U.S. [24–26] and Canada [27, 28]. In a study conducted across 45 U.S. states, 0.1%–24.9% lower vaccination was documented among Blacks vs. Whites, 0.0%–37.6% lower among Hispanics vs. Whites as of May 2021; state-level rate differences in Covid-19 vaccination were significantly associated with a metric of structural racism [22]. In another U.S. national study, eligible healthcare facilities and community pharmacies in counties with a higher composition of Black people were significantly less likely to provide Covid-19 vaccination [23].

Some of the racial/ethnic disparities in Covid-19 vaccination documented earlier in the pandemic have reportedly narrowed over time, attributed in part to the success of targeted public health interventions [25]. However, disparities continue to be evidenced in lower administration of booster doses among Black, Latinx, and American Indian populations versus Whites [29]. Consistent with the “inverse equity hypothesis,” with each innovation in Covid-19 vaccines, uptake is predicted to be greater among wealthier and more socially connected segments of the population, thereby increasing rather than reducing inequity [30, 31].

The World Health Organization (WHO) Strategic Advisory Group of Experts (SAGE) on Immunization defines vaccine hesitancy (VH) as “delay in acceptance or refusal of vaccination despite availability of vaccination services” [32,p.4161]. In addition to individual-level correlates of VH, SAGE [33] along with subsequent systematic reviews [34, 35] have drawn attention to the importance of vaccine availability and access, and factors that may be vaccine-specific. Systematic variations in Covid-19 vaccine coverage documented across and within countries and subpopulations support the need to identify and examine multilevel factors that contribute to Covid-19 VH and broader Covid-19 under-vaccination (e.g., due to inaccessibility, unavailability) [36]. The term “vaccine hesitancy” has also been criticized for being conceptually imprecise and employed as a catch-all label, in which structural inequities in Covid-19 vaccination are conflated with individual-level factors [22, 23, 37, 38]. This has important ramifications for vaccination: accurate classification and focus on factors that are driven by structural conditions shifts the onus from individual beliefs and cognitions as the primary or default target for effective interventions to institutional practices and policies [39–41].

Building on the SAGE “Vaccine Hesitancy Determinants Matrix,” which delineates contextual, individual and group, and vaccine/vaccination-specific influences [33]; recommendations to approach VH as a population-, context- and vaccine-specific phenomenon [33, 42]; and critiques of the lack of clarity in the definition and application of VH [39, 43, 44], we conducted a scoping review to identify and classify multilevel factors associated with Covid-19 VH and broader Covid-19 under-vaccination among marginalized populations in the U.S. and Canada.

## Methods

A scoping review was conducted given our aim of mapping the evidence landscape [45] on factors associated with Covid-19 VH and under-vaccination among marginalized populations. A scoping review protocol, including our search strategy and review process, has been published elsewhere [46]. We utilized the scoping review methodology described by Arksey and O’Malley [47], further developed by the Joanna Briggs Institute [48], with all results reported in accordance with PRISMA Extension for Scoping Reviews (PRISMA-ScR) guidelines [49].

### Research Question

This scoping review was guided by the question, “What are the determinants of Covid-19 ‘vaccine hesitancy’ reported among marginalized populations in the U.S. and Canada?”, with sub-questions, “What are the focal populations in studies of Covid-19 VH and under-vaccination among marginalized populations?” and “What are the contexts and determinants of Covid-19 VH and under-vaccination by structural, social and community, individual, and vaccine-specific domains?” [46].

### Information Sources and Search Strategy

We identified the following list of databases to search in consultation with a specialist research librarian: Medline, Embase, Cochrane CENTRAL, Cochrane Covid-19 Study Register, PsychINFO, Sociological Abstracts, and the International Bibliography of the Social Sciences (IBSS). We then developed a search string with keywords and synonyms related to vaccine hesitancy (i.e., “vaccine hesitancy”, “vaccine refusal”, “vaccine confidence”, “vaccine distrust”, “vaccine mistrust”, “vaccine barriers”) and Covid-19 (i.e., “corona virus” or “coronavirus” or “COVID” or “nCoV” along with adjacent terms “19” or “2019, or “SARS-CoV-2” or “SARS Coronavirus 2”, etc.). Supplementary Table 1 shows an example search string for Medline and Embase. We adapted the search string to accommodate differences in syntax and parameters among the selected databases.

### Study Selection Criteria

We developed inclusion and exclusion criteria prior to conducting the search for peer-reviewed articles published between January 1, 2020 and October 25, 2022: 1) populations in the U.S. or Canada; 2) adults ≥ 18 years; 3) focused on (> 50%) marginalized populations, 4) focused on Covid-19 vaccine hesitancy, refusal, confidence, mistrust, or barriers. Articles were excluded if they were 1) published prior to 2020; 2) not written in English; 3) focused on healthcare workers, staff, or students/interns; or 4) focused on children (< 18 years) or parents’ vaccination of children. We selected the beginning search date based on the WHO announcement on January 30, 2020, declaring Covid-19 a public health emergency of international concern [50].

We included the U.S. and Canada, which had similar approaches to managing Covid-19 and bilateral vaccine supply agreements. Marginalized populations were identified based on a review of populations particularly vulnerable to discrimination and exclusion in healthcare [51] or to broader adverse SDOH [13]. The populations include those marginalized in relation to race or ethnicity, sexual orientation or gender identity, physical or mental disability, people living with HIV, immigrants/refugees, people experiencing homelessness, justice-involved people, and people who use drugs, along with their intersections (e.g., Latinx immigrants). We anticipated that while studies focused on VH among the general population might indicate some factors (largely at individual and vaccine-specific levels) in common with marginalized populations (e.g., concerns about Covid-19 vaccine efficacy, side effects, and the influence of perceived risk of Covid-19 [52]), a focus on marginalized populations might provide more data on structural factors (e.g., systemic racism, institutional stigma, barriers in access to vaccination), facilitating a more refined understanding of VH and under-vaccination.

### Study Selection Process

Search results of peer-reviewed articles were uploaded into Covidence software (Veritas Health Innovation, Melbourne, Australia). Multiple research team discussions ensured consistent application of inclusion/exclusion criteria. Initially groups of two reviewers (among LR, TN, DD, KA, SF or ST) independently screened abstracts and titles until we attained inter-rater reliability of 90% [53]; this was achieved after two rounds of abstract review and team discussions to achieve consensus. Subsequently, in accordance with rapid review methods, one reviewer screened each abstract/title to determine inclusion or exclusion [54]. One reviewer (TN, DD, KA, SF or ST) then screened each full text for inclusion. Full-text articles designated for exclusion were then reviewed by a single arbitrator (PN), with discrepancies resolved by consensus among the research team [54, 55].

### Data Extraction and Synthesis

We abstracted data on publication characteristics (i.e., author[s], date, study sites/country, sample size, populations, methods) and factors identified in each of the four domains that influence VH or under-vaccination. We then reviewed the abstracted data using quantitative (i.e., frequency) analysis to describe study publication dates, country/regions, sample sizes, focal populations, research methods, and domains of factors associated with Covid-19 VH and under-vaccination. Based on coding each study by country, we identified if there were any systematic differences in VH or factors identified between the U.S. and Canada. We used qualitative (i.e., narrative) analysis to synthesize the evidence on VH and under-vaccination among each subpopulation.

Articles were coded as identifying a focal population if > 50% of the sample were from one marginalized group (i.e., African American/Black, Latinx, people with disabilities, etc.) and the analysis provided disaggregated data on that population. Articles with > 50% marginalized populations but without one focal subpopulation were coded as racial/ethnic minority (e.g., African Americans and Latinx combined were > 50% of the study sample). Articles describing samples with intersecting marginalized identities (e.g., Black or Latinx immigrants) were coded as “intersectional identities.”

After characterizing the population focus of each included article, we then allocated findings from articles coded as racial/ethnic minorities or intersectional identities as additional sources for thematic synthesis among each marginalized population for which disaggregated data were provided (i.e., one article could be a data source for several populations). Similarly, findings from focal (e.g., > 50% African American/Black) population articles that included disaggregated data for other marginalized subpopulations were included as additional sources for thematic synthesis among the respective subpopulation(s) (e.g., Latinx people).

Each publication was then coded using an a priori four-component framework by the domains of factors identified in relation to VH or under-vaccination. Our framework builds and expands on the “Vaccine Hesitancy Determinants Matrix” [33, 42]. While the Matrix helpfully acknowledges “contextual” influences in addition to individual-level factors in VH, several published critiques identify its under-emphasis and under-articulation of institutional and systemic barriers to vaccination [39–41]. For example, the Vaccine Hesitancy Determinants Matrix subsumes “health system and provider” together under “individual and group” influences, along with health beliefs and perceived risk; “historical” influences and “policies” are listed under contextual influences [33]. This may result in the lack of identification of structural racism and institutional mistrust (not specified in the Matrix) as barriers in health systems, and the unhelpful conflation of these and other structural factors with individual-level psychological influences, such as perceived risk [40, 41]. To that end, we used a social ecological model (SEM) [56] as a heuristic to identify and distinguish systemic-institutional, geographical, and other structural barriers in access to vaccination from social- and individual-level factors [39, 41, 57]. SEM has been widely used in the fields of public health and health promotion [58], and previously applied to conceptualize barriers to childhood vaccination among the general population [44].

The four domains of factors that influence vaccination comprised structural (e.g., structural racism and institutional mistrust, access/distance to vaccination site), social/community (e.g., family influence, trust in personal HCP, social proofing [i.e., based on actions of others in one’s community]), individual (e.g., perceived risk of infection, perceived Covid-19 threat, general vaccine beliefs), and Covid-19 vaccine-specific factors (e.g., vaccine safety, side effects). Factors were coded in a deductive process, derived directly from the studies, as well as inductively based on additional analysis in accordance with our a priori four-component framework. Coding of factors and their categorization were discussed in research team meetings, with any discrepancies resolved by consensus. One change to data extraction (but not study inclusion) from the initial protocol [46] is that we distinguished institutional and medical mistrust—distrust of healthcare systems, government, medical treatments, and/or the pharmaceutical industry—attributed to structural racism or other forms of structural stigma (structural) [40, 41], from trust in a personal HCP/clinic as a source of reliable vaccine information [59] (social/community).

## Results

**Fig. 1:**
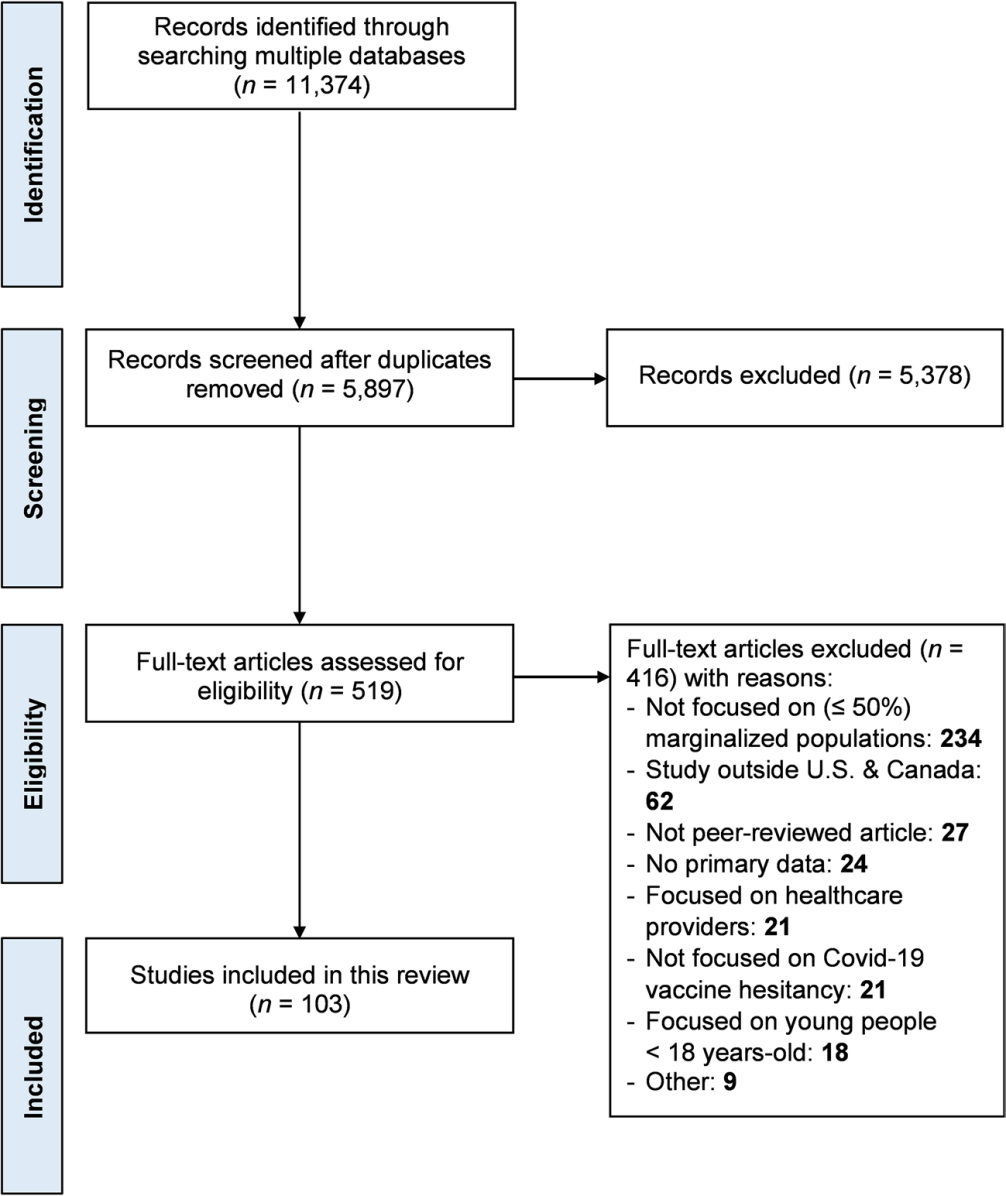
PRISMA flow diagram for scoping review of Covid-19 vaccine hesitancy and under-vaccination among marginalized populations

The search captured 11,374 peer-reviewed articles in total, with 5,897 abstracts screened after deduplication. We identified 519 full-text articles to be screened for eligibility, with a total of 103 peer-reviewed articles finally included [38, 60–161]. The PRISMA flow diagram shows the process of identifying relevant peer-reviewed journal articles (see Fig. 1).

### Description of Studies

**Fig. 2:**
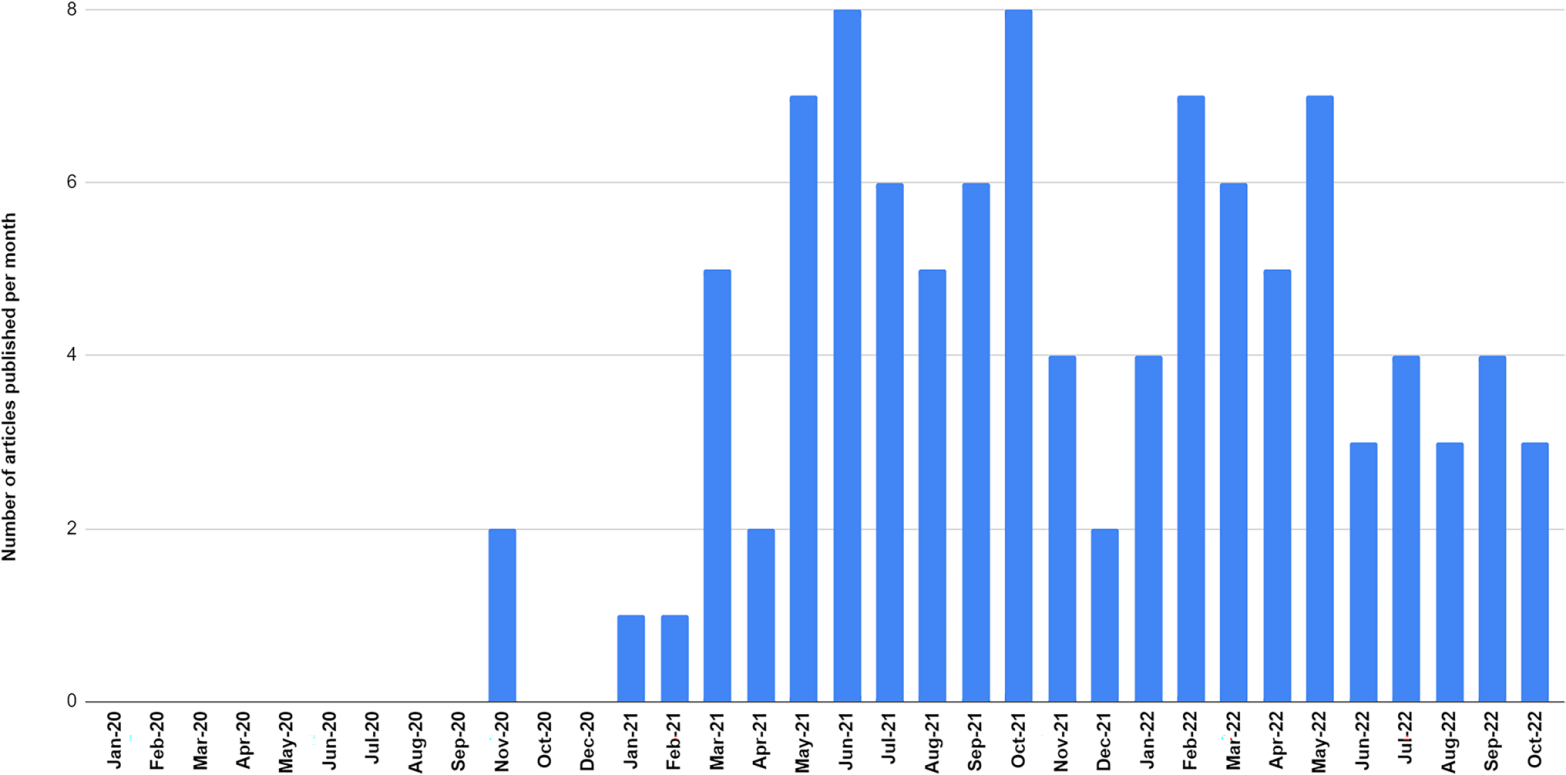
Publication trends on Covid-19 vaccine hesitancy among marginalized populations (*n* = 103)

Among the 103 included publications, the majority (n = 75, 72.8%) were quantitative studies, with 21.4% (n = 22) qualitative studies, as shown in Table 1. Sample sizes ranged from n = 10 to n = 164,283 (N = 442,211) with a median sample size of n = 346 (IQR = 110, 980). The vast majority of studies (n = 98; 95.1%) were conducted in the U.S., most of which (n = 34/98, 34.7%) were national studies, with 24/98 (24.5%) conducted in the West; 5/103 (4.9%) were conducted in Canada (see Table 1). The first article included was published in November 2020, with the majority (72.8%; n = 75) published from March 2021 through May 2022 (see Fig. 2).

**Table 1:** Characteristics of studies included in the scoping review of vaccine hesitancy and under-vaccination among marginalized populations in the U.S. and Canada

Study characteristics	Study references	n	%
Study design			
Quantitative	38,60,61,63–65,71–75,77,79–87,89,90,92–94,96,98–101,103,105–108,110–112,114–116,119,120,122, 124,125,128–137,141–148,150–154,156–160	75	72.8
Qualitative	66–70,76,78,88,91,95,97,102,104,113,117,121,123,127,138,140,149,161	22	21.4
Mixed methods	62,109,118,126,139,155	6	5.8
Country/Region			
US National	38,61,64,71,74,77,86,88–90,93,94,96,99–101,103,110,116,124,125,129,130,132,132,136,137,141,142,144,145,148,159,160	34	33.0
Midwest	65,75,81,84,97,117,120,126,151,153	10	9.7
Northeast	68,79,82,92,104,109,115,121,131,138,139,146,154	13	12.6
Southeast	63,69,70,83,87,105,106,108,112,122,123,134,155,156,161	15	14.6
Southwest	72,102	2	1.9
West	60,67,73,76,78,80,85,91,95,98,107,111,124,119,128,135,140,143,147,149,150,152,157,158	24	23.3
Canada	62,66,113,118,127	5	4.9
Population			
* Racialized*			
African American/Black	63,74,83,84,88,92,101,105,117,121–123,126,128,131,136,138,151,154,156,161 * Additional sources* (n = 39): 38,60,62,64,65,68,70,71,73,76–81,85–87,93,99,100,102–104,106,109, 114,119,120,125,133,137,141,142,144,145,150,153,159	21	20.4
Latinx	69,72,95,108,129,130,140,157 * Additional sources* (n = 21): 38,60,64,68,70,71,76–78,85,87,93,102,104,106,109,114,133,150,153,155	8	7.8
American Indian/Native Hawaiian/Indigenous	91,107 *Additional sources (n* = *5):* 66,78,98,102,118	2	1.9
Asian/Asian American	– *Additional sources (n* = *5):* 64,76,78,85,98	0	0.0
Arab American	61,110	2	1.9
Racial/ethnic minority	38,60,64–66,68,70,71,75–78,81,82,85–87,93,94,96,99,102,104,109,112,118,120,132,133,137,146,150,152,153	34	33.0
* Other marginalized populations*			
LGBTQ+	67,115,141,144,145 *Additional sources (n* = *4):* 62,69,103,155	5	4.9
People with disabilities	89,90,98,116,124,148,158	7	6.8
Justice-involved	80,97,100,113,114,127,142	7	6.8
People living with HIV	103,106,134 *Additional sources (n* = *4):* 73,79,141,144	3	2.9
People who use drugs	79,119,143,149,159 *Additional sources (n* = *1):* 98	5	4.9
People experiencing homelessness	111,135,147 *Additional sources (n* = *2):* 62,67	3	2.9
Immigrants and refugees	139,160 *Additional sources (n* = *2):* 109,125	2	1.9
Intersectional identities	62,73,125,155	4	3.9

Fourteen marginalized subpopulations were identified in the included articles (see Table 1). A plurality (n = 34, 33.0%) focused on racial/ethnic minorities as a whole, with the largest single population focus on African American/Black people, representing one-fifth (n = 21, 20.4%) of the overall focal articles. Latinx people accounted for the next largest category, with 8 articles (7.8%), followed by 7 (6.8%) each on people with disabilities and justice-involved people (See Table 1). Data allocated as additional sources from “racial/ethnic minority” and “intersectional identities” categories, along with disaggregated findings from focal population articles, predominantly addressed African American/Black populations (n = 39/82 additional) and Latinx populations (n = 21/82 additional). With no focal articles on Asians/Asian Americans, the 5 additional sources provided the only data on this population. Additional sources also provided substantial additional data for American Indians/Indigenous peoples, LGBTQ+ people, and people living with HIV.

**Fig. 3:**
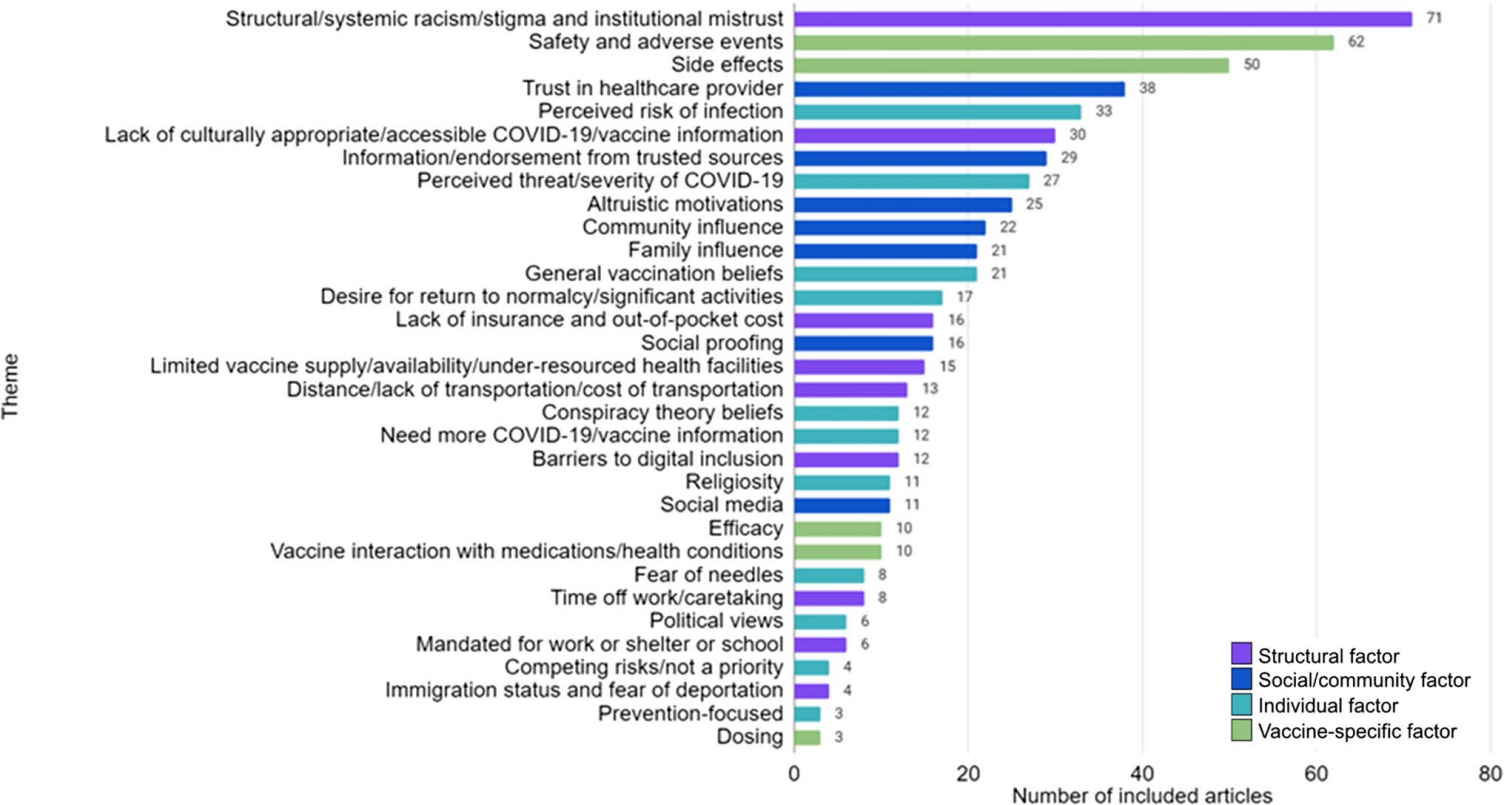
Factors associated with Covid-19 vaccine hesitancy and under-vaccination among marginalized populations by structural, social/community, individual, and vaccine-specific domains

Thirty-two factors were identified as influences on Covid-19 VH or under-vaccination across the 103 included publications. Among these, 9 structural, 7 social/community, 11 individual, and 5 vaccine-specific factors were identified (see Fig. 3). The most prevalent factor overall was structural racism/stigma and institutional mistrust (structural) (n = 71), followed by safety/adverse events (vaccine-specific) (n = 62), side effects (vaccine-specific) (n = 50), trust in an individual HCP (social/community) (n = 38), and perceived risk of infection (individual) (n = 33). Overall, structural factors predominated (n = 175), followed by social/community (n = 162), individual (n = 154), and vaccine-specific factors (n = 135) across marginalized populations.

**Fig. 4:**
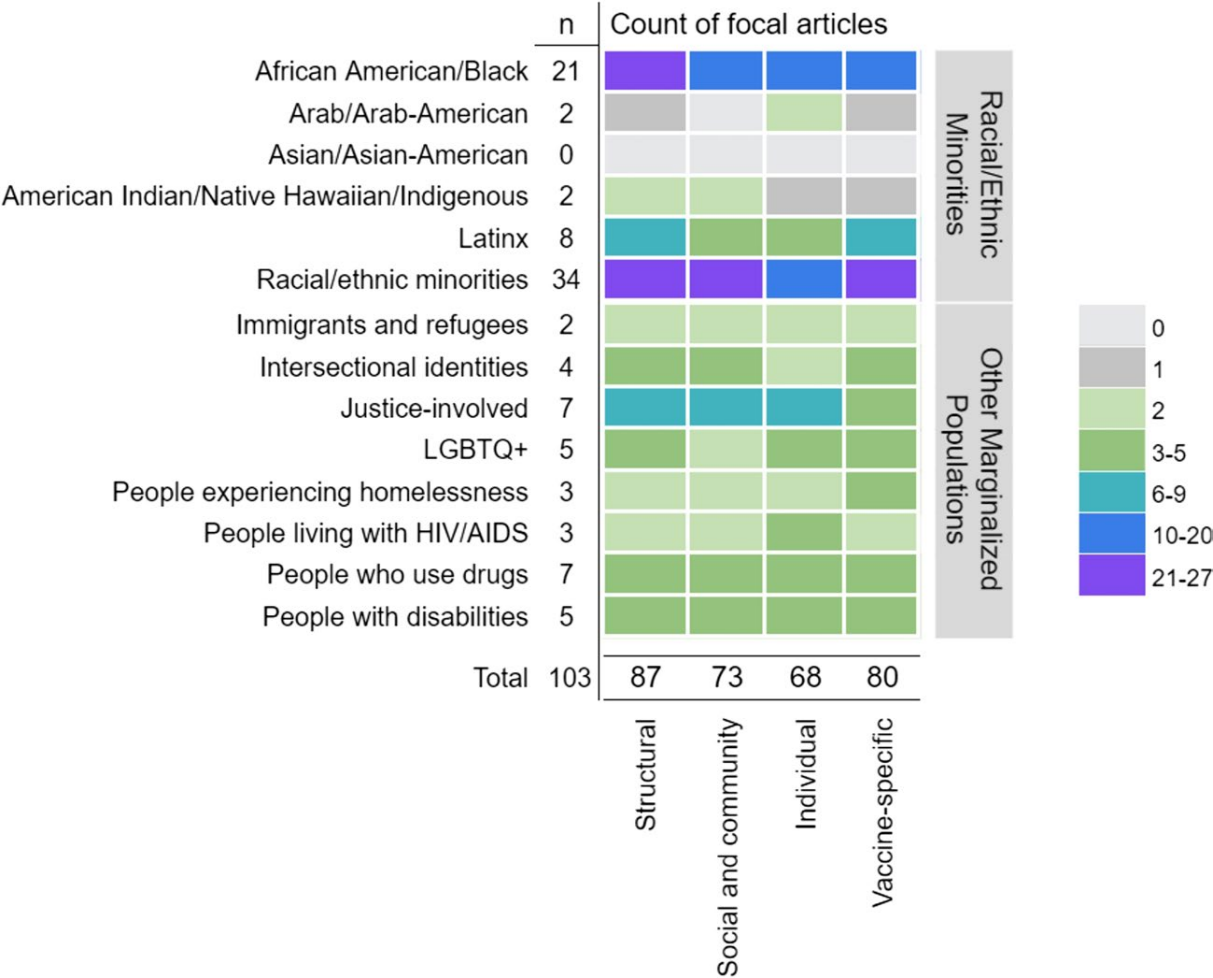
Domains of Covid-19 vaccine hesitancy and under-vaccination by focal population

Finally, we identified the number of focal articles on each subpopulation that addressed each of the 4 domains of VH/under-vaccination (see Fig. 4). Notably, 100% (21/21) of focal articles on African American/Black people identified structural factors associated with VH and under-vaccination, with structural racism and institutional mistrust the predominant theme. Although comprising considerably less publications, 100% of the focal articles addressing American Indian/Native Hawaiian/Indigenous peoples (2/2), immigrants and refugees (2/2), and justice-involved people (7/7) identified structural factors, along with a substantial majority of those focused on Latinx (87.5%; 7/8) and LGBTQ+ people (80%; 4/5).

### Black and African American People

A plurality of publications on VH—(n = 60); 21 focal articles, 39 additional sources—addressed Covid-19 VH/under-vaccination among Black/African American people. The impact of structural racism and institutional mistrust pervaded the structural level, with 48 sources describing negative impacts on Covid-19 vaccination among Black/African Americans [38, 62, 64, 65, 68, 70–72, 74, 76–78, 80, 81, 83, 84, 86–88, 92, 99, 101–106, 109, 117, 119–123, 125, 126, 128, 131, 136–138, 142, 144, 145, 151, 156, 159, 161]. Two-thirds (n = 41/60) of sources identified structural or systemic racism as the cause of medical mistrust. The majority of these addressed historical legacies of medical racism and health inequities, such as fears of being experimented on with Covid-19 vaccines without consent, “based on…knowledge of historic mistreatment and unethical research practices that adversely impacted black patients and participants” [121,p.1786], including the legacy of the Tuskegee Study of untreated syphilis [78, 88, 102, 104, 109, 123, 125, 126, 128, 138, 161]. One-third (n = 20/60) specifically acknowledged present-day barriers to healthcare and Covid-19 vaccines for Black/African Americans, including unemployment, lack of insurance, housing insecurity, and challenges in traveling to vaccination sites [60, 68, 70, 74, 76, 78, 83, 84, 86, 88, 101, 106, 109, 122, 123, 126, 133, 138, 151, 154]. Several studies discussed lack of clear and consistent information regarding Covid-19 vaccines and vaccination processes as contributing to under-vaccination [76, 88, 102, 109, 125, 138, 161].

At the social/community level, 19 sources addressed the role of trust in HCPs as sources of Covid-19 vaccination recommendations [38, 64, 73, 77, 84, 87, 88, 93, 102, 105, 106, 109, 121, 128, 131, 137, 138, 153, 161]. Characteristic of several studies, in a U.S. population-based online survey (n = 1950), “Blacks and Hispanics were more likely than Whites to state that it was ‘extremely’ important that a medical professional of their same race/ethnicity endorse the vaccine before they took it” [38,p.8]. Eight studies addressed the influence of families and communities on Covid-19 vaccination [68, 74, 92, 102, 109, 117, 136, 138], and further that “governmental agencies are less well-received as information sources compared to friends or family” [61,p.1103]. Social proofing was identified as an influence on Covid-19 vaccination, with individuals reporting greater willingness “…now that millions of people have been vaccinated…” [138,p.1311]. However, in one study, family influence promoted VH by casting doubt on public health information, rather than motivating vaccination [136]. Additionally, 7 sources addressed the influence of faith-based organizations and pastors on Covid-19 vaccination among Black/African American people [60, 87, 102, 109, 117, 123, 161]; e.g., “pastors can be a big influence for people to get vaccinated. They have to continue to talk about and encourage people” [123,p.4].

At the individual level, 7 studies identified negative impacts of religious beliefs, including preference for ‘natural’ medicines over vaccines and general vaccine aversion, on Covid-19 vaccination [117, 119, 121, 126, 131, 136, 138]. Perceived risk and perceived threat of Covid-19 also influenced VH/vaccination [82, 84, 85, 101, 102, 117, 122, 126, 131, 138]; for example, among a predominantly African American and Latinx sample in the northeastern U.S. (n = 203), “those who believed…that the virus is not serious (OR: 8.28, 95% CI: 1.11, 61.8) showed greater odds of hesitancy” [82,p.2]. Vaccine-specific concerns, described in 30 studies, included vaccine safety/adverse effects, concerns about Covid-19 vaccine interactions with other medications or conditions, and uncertain efficacy [38, 65, 70, 73, 74, 76–78, 84, 85, 87, 88, 92, 101, 102, 104–106, 109, 117, 119–121, 124, 126, 131, 136, 138, 154, 161]. Several sources described the importance of evidence of Covid-19 vaccine safety and efficacy specific to Black/African Americans, such as “any data that’s connected with some of the conditions that are prevalent in our community” [78,p.8].

### Latinx People

Eight publications were focused on Covid-19 vaccination among Latinx people, complemented by 21 additional sources providing disaggregated data. Of these 29 articles, 13 addressed the structural level, including negative impacts of institutional and medical mistrust [68, 71, 76, 82, 87, 95, 108, 109, 140, 155, 157], fear of being used as “guinea pigs (*conejillos de indias*)” and fear of being sterilized [95]. Lack of access to clear information about Covid-19 vaccines and vaccination, particularly in one’s primary language; complex online vaccination sign-up procedures; lack of insurance coverage; and lack of transportation to vaccination sites posed structural barriers to vaccination [68, 70, 76, 78, 85, 95, 102, 104, 106, 109, 130, 133, 140, 155]. Eight studies underscored the importance of tailored and reliable vaccine education and information [68–70, 76, 102, 104, 109, 129], noting that gaps may be filled by non-expert sources—a “cocktail of information, lots of very bad information” [69,p.8]: e.g., “[a family member] doesn’t speak English…so, a lot of the information that he may have heard about the vaccine and Covid probably came off of Univision and Telemundo” [68,p.6].

At the social/community level, 13 studies described the important role of personal HCPs in Covid-19 vaccination among Latinx communities [38, 60, 64, 69, 76, 77, 87, 102, 106, 114, 153, 155, 157], including endorsement from an HCP of one’s own ethnicity [38, 76]. Eight studies addressed altruism, specifically in wanting to protect one’s community, as motivating Covid-19 vaccination [69, 78, 95, 102, 106, 109, 139, 155]. Families and close communities were also described as playing an important role in Covid-19 vaccination knowledge and decision-making [68, 69, 95, 102, 109, 157]: in a qualitative study of Latinx families (n = 46), “79% [n = 19/24] of the youth had discussed health with their families for an average of 50 min [per week]” [95,p.753].

At the individual level, religious [102, 130, 140] and general vaccination beliefs [140, 155] influenced Covid-19 VH. Further, a desire for a return to “normalcy” [69, 76, 95, 102, 140, 155], including cultural familial traditions [140] and mitigating economic precarity [76, 95, 140], were described as motivators for vaccination: “The fear and isolation they experienced placed them in precarious situations in which some ‘only had cereal to eat’ and others were on the brink of losing their housing. These families saw the vaccine as a way to safely return to work and recover some socioeconomic stability” [95,p.750]. Pervasive Covid-19 vaccine-specific concerns were described around safety, side effects, vaccine interactions with other medications, and efficacy [38, 69, 70, 76, 77, 85, 87, 95, 102, 104, 106, 108, 109, 130, 140, 153, 155, 157].

### American Indians, Native Hawaiians, and Indigenous Peoples

Structural barriers to Covid-19 vaccination among American Indians, Native Hawaiians, or Indigenous peoples were reported due to institutional mistrust, specifically distrust of government and a desire for autonomy—“to be respected” [78,p.8]. Challenges in access emerged around digital technology and the internet, lack of quality translations and culturally tailored information, and lack of employment benefits, insurance, and transportation to distant vaccination sites [66, 78, 91, 102, 107, 118]. Distrust was reported due to the politicization of vaccination, as well as structural barriers for older adults: “Some elders…they’re houseless…there’s no cellphone, they ride public transportation. How is it going to get distributed [to them]…the most vulnerable…it feels like our culture always gets the shitty end of the stick” [78,p.6]. A First Nations (Indigenous) participant in a Canadian study highlighted differential access to information in their preferred language: “provincial and federal governments are pumping out information in like…really common languages, but they weren’t pumping it out in Oji-Cree” [66,p.5].

At the social/community level, the role of family and community in Covid-19 vaccination among American Indians/Indigenous peoples included community and Elder’s involvement in decision-making and altruistic motivations to protect one’s community [91, 102, 118]: “Elders in the family were just like, ‘get the vaccine now, now, now!’ They pushed us all to do it for them” [102,p.147]. Community HCPs emerged as trusted sources of information able to facilitate vaccination [91, 100]: “our Native physicians carry a lot of weight as being trusted leaders and healers in the community. And maybe even some of our trusted Medicine people to also support [vaccination] and encourage it” [102,p.148].

Covid-19 vaccine-specific concerns arose around side effects, efficacy, and multiple doses [78, 91, 98, 118]; the latter was reported as a challenge for Native families due to the several costly trips to distant vaccination sites outside of their community [78].

### Asian People

At the structural level, barriers to vaccination among Asian people included difficulties in accessing reliable translations and messengers of Covid-19 vaccine information [76, 78]. Beyond linguistic translation, a Filipinx participant explained, “In terms of translators…maybe those who may not be of the same culture or the way that they’re explaining the medical terminology may be intimidating” [78,p.6]. Institutional mistrust was identified as negatively impacting Covid-19 vaccination [78], with trust in personal HCPs as sources of vaccine information positively associated with Covid-19 vaccination [64, 76]. Notably, a Canadian study described experiences of discrimination among Asian people during the pandemic as influencing vaccination. Perceived necessity of being vaccinated was attributed to being deemed responsible for the pandemic, as expressed by a Chinese woman: “like I was getting blamed [for Covid-19] because of my skin color”, as people would “give you dirty looks, as if you caused it” [118,p.8].

Vaccine-specific concerns arose around safety, side effects, and efficacy [76, 78, 98], along with needs for population-specific evidence: “There has been a lot of concerns in my family on how the vaccine works for people with heart disease, which really affects…a lot of the Filipino community, and also those with respiratory diseases” [78,p.6].

### Arab Americans

At the structural level, institutional mistrust emerged among Arab Americans, with 70% (n = 73/105) of unvaccinated individuals in one study (N = 1746) reporting “distrust in the healthcare system, vaccines, and the government” [110,p.6]. At the individual level, gender and religiosity were associated with Covid-19 VH: in a national survey of Arab Americans (n = 638), “of those unlikely to receive the vaccine, 72.9% [n = 35/48] were women and 85.4% reported moderate to high religiosity [n = 41/48] (p < 0.01)” [61,p.2188]. Covid-19 vaccine-specific factors arose in concerns about safety and side effects [110].

### LGBTQ+ People

At the structural level, several studies addressed structural transphobia and homophobia as engendering institutional and medical mistrust, based on historical and ongoing discrimination, and trauma experienced both within and outside the healthcare system by LGBTQ+ people [62, 67, 144, 145]. In particular, misgendering and fear of emotional or physical violence emerged as barriers to Covid-19 vaccination among transgender people, as in a Los Angeles-based study: “Transwomen are afraid that they are going to be beat…[afraid] for their lives…of being attacked…to get the vaccine” [67,p.406]. Limited access to clear and culturally tailored messaging for LGBTQ+ people and those marginalized across multiple demographic intersections, such as LGBTQ+ and racial/ethnic minority identities (e.g., transgender Latinx), also posed barriers to Covid-19 vaccination [62, 67, 69, 155]. Moreover, intersectional challenges arose as a result of economic and social marginalization, which produces adverse SDOH within LGBTQ+ communities, such as among both LGBTQ+ youth and LGBTQ+ older people experiencing homelessness [62, 67, 115, 155]. Lack of transportation, lack of insurance coverage, and complex online vaccination booking systems also emerged as barriers in access to vaccination [62, 115, 155]. Loss of employment and housing insecurity were described as factors that underscored the threat of the pandemic among LGBTQ+ people, thus motivating vaccination [141]. 

At the social and community level, altruistic motivations [69, 115, 145, 155] positively influenced Covid-19 vaccination, while social concerns about others’ perceptions [145] negatively influenced Covid-19 vaccination. Individual-level factors impacting vaccination were described in the perceived threat of Covid-19 [62, 69, 141, 155]: “negative experiences, along with the reported high levels of perception of the seriousness of Covid-19 infection, are likely driving the high levels of willingness to be vaccinated” [141,p.9]. Covid-19 vaccine-specific concerns included safety, side effects and efficacy [62, 115, 145, 155].

### People with Disabilities

Among people with disabilities (PWD), at the structural level mixed findings emerged on the impact of institutional trust/mistrust: three studies reported a positive influence (e.g., “greater trust in the CDC”) [90,p.5, 89, 148]. “Inaccessibility of public transportation” [148,p.7] and widespread reliance on digital technology during the pandemic posed structural barriers in access to Covid-19 vaccination for PWD [116, 124].

At the social and community level, having a trusted HCP/clinic mitigated VH [89, 124, 148, 158]. For example, among people living with multiple sclerosis (MS), MS-specialist HCPs and the National MS Society were deemed trusted sources: “well-positioned to provide accurate education about Covid-19 disease and vaccine considerations…specific to the MS population as well as their individual patients” [89,p.3].

At the individual level, perceived risk and threat of Covid-19 were positively associated with Covid-19 vaccination among PWD, including individuals with chronic diseases and multiple comorbidities [89, 90, 98, 116, 124, 158]. Covid-19 vaccine-specific concerns included safety/adverse effects, speed of vaccine development, and efficacy [90, 98, 116, 124, 148, 158]. In a national online survey of people with MS (n = 701), in addition to “safety and efficacy…the top Covid-19 vaccination concerns”, apprehension arose around “the effect of the vaccine on MS symptoms and implications for [immunosuppressive] DMT [disease-modifying therapy] use” [148,p.1078].

### Justice-Involved People

Justice-involved individuals include currently or formerly convicted or incarcerated people. At the structural level, most studies addressed institutional mistrust as fomenting Covid-19 VH among individuals in detention facilities [80, 97, 113, 114, 127, 142], “based on their interactions with law enforcement or the justice system or their experiences with institutional racism” [142,p.476]. A study with incarcerated women described “a direct correlation between trust in the pharmaceutical industry and willingness to receive the vaccine”: “I’ve watched too many people die because of pharmaceuticals… So I don’t know…pharmaceutical companies just see a lot of big money in there” [97,p.895]. Lack of accessible and reliable information on Covid-19 vaccines contributed to VH within prisons [80, 113, 127]: “[The information on the vaccine] is not disseminated. It’s not packaged properly. And the way that things are in prisons, we hardly receive any information at all” [113,p.4]. One study described mitigation of structural barriers through tailored Covid-19 vaccine education and “early access to vaccine through a dedicated federal allocation” for incarcerated individuals and facility staff [80, p.5889].

At the social and community level, family and community influence were associated with Covid-19 VH [97, 114, 127] among justice-involved individuals in the absence of tailored public health messaging: “The probable low health and informational literacy of these women leave them vulnerable to influence from family and friends, many sharing and promoting disinformation and conspiracy theories frequently shared by homegrown anti-vaccine organizations” [97,p.895]. However, family and community were also described as mitigating Covid-19 VH: “I think what made my decision is that I spoke to my family and we had many conversations regarding the pros and cons…” [113,p.6]. Social proofing in closed systems also motivated Covid-19 vaccination [100, 113, 142]: “some people may have been encouraged to accept vaccination because they could see their peers being vaccinated” [100,p.5889]. Nevertheless, ambivalence toward jail health staff complicated their influence [113, 114]: “trust in jail health staff was much lower than trust in one’s doctor outside of jail, although both were associated with higher vaccine acceptance” [114,p.3]—“When you were in need, they walked away from you. But now they wanna give you a COVID shot?” [127,p.9]

At the individual level, mixed findings emerged about perceived risk and threat of Covid-19 among justice-involved people [80, 97, 100, 113, 127, 142]. In a 4-state study in correctional and detention facilities, 18.9% (n = 344/1823) of participants (n = 1823/5110) who would refuse Covid-19 vaccination “did not perceive themselves to be at risk for Covid-19 or thought vaccination was unnecessary” [127,p.475]. Low perceived risk was associated with VH: “Getting the COVID-19 vaccine doesn’t make sense for someone in my position…when I don’t interact with anybody in the community, I don’t talk or be around anybody in the community…I don’t need it right now” [127,p.5]. In contrast, high perceived risk was reported due to incarceration: “Because it’s such a close-knit community and there’s no real social distancing. It’s hard to socially distance when you live in the same living unit with somebody. [Covid-19] would catch like wildfire. [The vaccine] would stop me from getting it, and would stop you from spreading it” [113, p.6]. Covid-19 vaccine-specific concerns arose around vaccine safety, side effects, and efficacy [97, 113, 114, 127, 142].

#### People Living with HIV

At the structural level, institutional and medical mistrust were associated with VH among people living with HIV (PLHIV) [73, 103, 106], including those with multiple marginalized identities who experienced intersectional racism, homophobia, and HIV stigma. A study with HIV-positive and predominantly sexual minority Black Americans (n = 101) indicated that “The most prevalent general mistrust beliefs (endorsed by about half or more than half) concerned withholding information or a lack of honesty by the government,” which was correlated with VH [73,p.203]. A study with Black and Latinx PLHIV described unemployment being exacerbated by the pandemic, with negative impacts on access to healthcare and Covid-19 vaccination [106]. A national study of PLHIV identified intersectional challenges that disadvantaged “heterosexual men living with HIV” who “may have less access to health resources, as a large proportion of HIV services are oriented toward sexual minority men…straight men may not tap into the same networks that provide trusted information about COVID-19” [103,p.42]. At the social and community level, personal HCPs as trusted sources of information mitigated Covid-19 VH [73, 79, 106, 134]: “Lower hesitancy scores were present in participants who reported that they would get vaccinated if their provider recommended it” [134,p.98].

At the individual level, perceived risk and perceived threat were associated with Covid-19 vaccination [103, 134, 141]: “…HIV-positive men may have more concerns about the seriousness and perceived impact of Covid-19 infection on their health” [141,p.9]. Experiences in the HIV pandemic were described as mitigating VH: “For youth living with HIV and youth highly engaged in HIV prevention, vaccine acceptance and engaging in protective behaviours for COVID-19 may be related to lessons gleaned from understanding the importance of behaviour change and adaptation in response to the HIV pandemic” [144,p.9]. Covid-19 vaccine-specific concerns arose around vaccine safety, side effects, and efficacy [73, 106, 134, 144], with some PLHIV expressing concerns that their health condition may make Covid-19 vaccination unsafe [134].

### People who use Drugs

At the structural level, barriers to accessing healthcare and Covid-19 vaccines as well as institutional mistrust among people who use drugs (PWUD) were associated with lower Covid-19 vaccination [98, 119, 143, 149]: “I wouldn’t even know where to go [to] research [vaccines]…I can look up things online, but there are so many different places” [149,p.3]. Vulnerability to misinformation was described among PWUD amid institutional mistrust: “As a marginalized population, patients with illicit drug use are often detached from and mistrust the healthcare system—by extension, these patients may be more reliant on illegitimate information sources” [98,p.803]. In another study with PWUD, “a majority of participants [85%, n = 393/550]…endorsed at least 1 conspiracy theory related to COVID-19 or vaccines” [143,p.728]. At the social and community level, benefits of vaccine-related information from trusted sources, including personal HCP, community health workers, and peers were reported—having “someone like us [say] that it did actually help” [149,p.4,159]. Obversely, among PWUD (n = 550) in the southwest border region, “Vaccine-hesitant participants were more likely to identify social media as their primary source of COVID-19-related information (21.1% vs. 8%, p < 0.001)” [143,p.728].

At the individual level, low perceived threat of Covid-19 infection was associated with VH [143, 149]: “if I were to get COVID, it’s going to be very mild, so to put something extra in my body isn’t worth it…this flu…SARS thing isn’t going to be that detrimental to me [149,p.2]; however, understanding that the disease is “real, dangerous” influenced individuals to “take any medication or vaccine to prevent it” [149,p.3]. Covid-19 vaccine-specific factors arose around safety and efficacy concerns that may negatively impact uptake [79, 119, 143, 149].

### People Experiencing Homelessness

Among people experiencing homelessness (PEH), at the structural level, institutional mistrust of government and healthcare systems was associated with VH [62, 111, 135]. In one study, Covid-19 VH was described as a function of “a more general distrust of systems (e.g., health and homeless services) that are not designated to meet their needs” [110,p.6]. In a study with LGBTQ+ youth experiencing homelessness, a participant reported “Homeless people are almost never viewed as actual human beings” [62,p.7]. Limited access to clear and culturally tailored messaging, lack of transportation, and complex online vaccination booking systems created barriers in access to Covid-19 vaccination [62, 111]: “the provincial government did a terrible job trying to explain how it all worked and whatnot. And I had to take on the task of booking appointments just because everyone else found it so difficult to try and navigate” [62,p.8].

At the social/community level, family, community, social media, and community housing staff exerted an influence on vaccination [62, 111, 135]. Among PEH surveyed in Los Angeles (n = 1306), one-fifth identified “protect others (community, friends, family)” and “[housing] staff recommended it”, respectively, as reasons for vaccine readiness [135,p.598]. However, trusting information received from friends, family, and social media rather than “official sources” was associated with greater Covid-19 VH [111,p.1]

At the individual level, general vaccine aversion and low Covid-19 threat perception [111], were associated with VH among PEH. However, perceived threat was described in a structural context in which vaccination may not be a priority [62, 67, 135]: “When someone is unhoused, it’s almost like survival trumps getting vaccinated” [67,p.406]. Vaccine-specific concerns emerged around safety, side effects, and efficacy [62, 111, 135, 147].

### Immigrants and Refugees

At the structural level, barriers to healthcare and Covid-19 vaccine access among immigrants and refugees negatively impacted vaccination [109, 125, 139, 160]. Among Latinx immigrant families, fear of immigration enforcement was a barrier to accessing services during the pandemic—“[Undocumented families] they’re the ones being infected at a higher rate, they’re the ones being evicted. They’re the ones that are being rounded up by ICE [Immigration and Customs Enforcement]” [109,p.399]. Limited access to information in one’s preferred language, lack of reliable information from trusted sources, constraints in access to the internet and digital technology, and lack of transportation and employment benefits (i.e., paid sick days) were identified as barriers to Covid-19 vaccination [109, 125, 139, 160]. An immigrant from West Africa explained, “Expecting people to log in to schedule appointments is not reasonable—you’ve lost them at ‘login’” [109,p.402].

At the social and community level, receiving Covid-19 vaccine-related information from a trusted source, such as family, friends, personal HCPs, or community leaders, positively influenced Covid-19 vaccination [109, 139]. Multigenerational communication among some immigrant families promoted Covid-19 vaccine information sharing, with a “respect your elders” approach employed to influence vaccination [109,p.403]. Family influence on altruistic motivations was demonstrated in a national online survey of refugees (n = 435): among those who reported intention to receive a Covid-19 vaccine, nearly two-thirds (65.0%; n = 199/306) indicated that it was motivated in part to protect their family members [160]. At the individual level, religious beliefs—“concerns that the vaccine is religiously prohibited” [139,p.1233]—were a barrier among some refugees. Covid-19 vaccine-specific concerns about vaccine safety and efficacy were associated with VH [109, 125, 139, 160]: among those refugees (29.7%, n = 129/435) reporting no intention or being unsure of accepting Covid-19 vaccination, 71.3% (n = 92/129) indicated concerns about side effects [160].

## Discussion

This scoping review of Covid-19 vaccine hesitancy and broader under-vaccination identifies multilevel determinants of Covid-19 vaccination across marginalized populations in the U.S. and Canada. Overall, this review provides evidence of a preponderance of structural-level factors that produce pervasive barriers in access to Covid-19 vaccination among marginalized communities. These include barriers in access to Covid-19 vaccination due to lack of vaccine availability at familiar/local community venues, lack of transportation to distant sites, lack of paid sick days, and barriers in access due to being unemployed, unstably housed, uninsured/underinsured, and the digital divide (i.e., lack of access to digital technology and the internet, and the technical and financial ability to utilize it [162]). These barriers were compounded by lack of culturally tailored and linguistically appropriate information and access to trusted HCPs from one’s community. Collectively, these findings suggest that approaches to under-vaccination among marginalized populations that focus predominantly on individual-level psychological and vaccine-specific factors may fail to capture the breadth and impact of determinants of Covid-19 vaccination.

### Structural Stigma, Racism, and Institutional Mistrust

Importantly, we identified extensive evidence for structural racism and other forms of structural stigma as sources of institutional and medical mistrust [162–165] associated with VH [40, 41]. Structural stigma based on racism, transphobia, homophobia, ableism, xenophobia, and other ideologies that support marginalization, engendered mistrust of government, public health, healthcare systems, and the pharmaceutical industry across various populations—including African American, Latinx, American Indian, and LGBTQ+ people, and their demographic intersections. However, many studies attributed institutional mistrust only to historical discrimination and past unethical medical research absent any invocation of present-day structural racism. Moreover, several studies while identifying “medical mistrust” omitted mention of structural or institutional racism or stigma, in effect treating mistrust produced by institutional policies and practices as an individual-level psychological factor [40, 41]. Institutional mistrust was also evidenced among JI populations, PEH, and PWUD due to generally negative and dehumanizing experiences with government and healthcare institutions. This potent structural context is largely unaccounted for in most VH research. A pre-Covid-19 systematic review similarly revealed trust in vaccination to be an ill-defined and inconsistently measured construct across studies [34]. The inconsistent conceptualization and assessment of trust emerges as particularly problematic in the context of Covid-19 vaccination among marginalized communities given the complex and multilevel impact of trust on Covid-19 vaccination—in structural, social/community, and vaccine-specific domains.

### Trust in a Healthcare Provider

Trust in a personal HCP or clinic emerged as the most prevalent influence at the social/community level. Having a trusted HCP, sometimes but not uniformly identified as one with shared race or ethnicity, or a familiar source of primary care, mitigated Covid-19 VH [38, 76, 102, 109, 128, 138]. Notably, trust in a HCP contributed to attenuating VH even amid broader institutional mistrust of healthcare systems and government—evidence for the importance of distinguishing among different types of trust in vaccination [34]. Personal HCPs and local community venues often assuaged concerns about vaccine safety and novelty, thereby reducing VH, indicating a crucial role for primary care [166]. In this light, the absence of information from trusted sources tailored to address healthcare needs experienced as most relevant to one’s own community—specifically reported among Black and Filipinx populations, PWD, and PLHA—and lack of availability of Covid-19 and vaccine information in one’s primary language—reported among Latinx, American Indian/Indigenous people, Asian, and immigrant/refugee populations—reveal substantial missed opportunities to facilitate Covid-19 vaccination among marginalized communities. Access to Covid-19 vaccination from a known or trusted HCP or clinic may be particularly important for individuals from marginalized communities, who are understandably more predisposed towards institutional mistrust due to negative interactions with the healthcare system and other societal institutions, and more vulnerable to ongoing discrimination in healthcare [167, 168].

### Conflation of Structural, Social/Community, and Individual Factors

Overall, structural factors—documented in multifaceted barriers in access to Covid-19 vaccination and vaccine information, and structural racism and other forms of structural stigma in healthcare systems and government institutions—were sometimes conflated with individual (e.g., perceived threat of Covid-19, perceived risk of transmission, general vaccine beliefs) and social/community factors (e.g., family influence, endorsement from a trusted HCP). While culturally tailored interventions to address individual and social/community factors may help to mitigate or resolve “decisional conflict” that is manifested in VH [43], they are unlikely to foster solutions that effectively address structural and systemic barriers in access to Covid-19 vaccination [39, 164, 169]. In fact, the attribution of structural-level barriers to individual volition and community deficits may not only fail to support effective interventions, but may counterproductively exacerbate alienation and mistrust among populations already marginalized due to ongoing systemic discrimination, stigma, and adverse SDOH [167]. The phenomenon of institutional mistrust has been aptly described as an adaptive response to an ongoing legacy of medical racism [41].

### Population- and Context-Specific Influences on Vaccine Hesitancy

Notably, some of the same factors showed negative or positive associations with VH depending on the population and context. For example, family influence mitigated Covid-19 VH among Latinx communities, immigrant/refugee populations, and their intersections, through familial sharing of health information and motivations to protect one’s family. However, among several other populations, the context of structural racism and institutional mistrust coupled with the virtual vacuum created by the absence of access to culturally tailored public health information rendered family influence a liability, resulting in promulgation of conspiracy theories and mistrust of Covid-19 vaccines among some African Americans [136], PEH [111], and JI persons [98]. These mixed findings underscore the importance of approaching VH as a population-specific and context-dependent phenomenon [33, 34], in addition to the imperative of disentangling structural factors from individual-level and social/community concerns [170]. Absent a context-dependent understanding, the same intervention applied to what might be understood as the same factor (e.g., family influence) may exert divergent effects on Covid-19 VH across different populations.

### Population- and Culture-Specific Facilitators of Covid-19 Vaccination

Importantly, several facilitators of Covid-19 vaccination emerged despite simultaneous constraints. Findings in common across PWD and PLHIV—among whom one might reasonably expect heightened vaccination concerns due to underlying comorbidities and potential medication interactions—included benefits of regular contact with a personal HCP or clinic; this promoted vaccine confidence through interpersonal trust and belief in one’s HCP’s understanding of and ability to address population-specific concerns. Similarly, despite trenchant institutional mistrust among American Indians and African Americans, Native physicians and traditional healers, and the Black church and faith-based leaders were highly influential in assuaging VH and promoting Covid-19 vaccination. Moreover, proactive interventions for some justice-involved populations involving tailored Covid-19 and vaccine education in tandem with policy decisions to prioritize Covid-19 vaccination in prisons mitigated VH in an otherwise fraught context, thereby effectively promoting vaccination [100].

We also identified evidence of strengths born of intersectional identities. Among Latinx immigrant/refugee populations who reported structural barriers owing to potential legal repercussions of their seeking vaccination, and healthcare more broadly, cultural norms supporting intergenerational sharing of health information and altruistic motivations to protect one’s community mitigated VH [157]. For Latinx sexual minority men, altruistic values within Latinx cultures and LGBTQ+ communities synergistically mitigated VH [155].

It is crucial that interventions approach culture beyond a deficit model to leverage community assets and sociocultural facilitators [171] that support Covid-19 vaccination. To that end, increased research on VH/under-vaccination among intersectionally marginalized communities, such as in-depth qualitative subpopulation studies following broader surveys, is warranted to address heightened vulnerabilities and mobilize population-specific strengths.

### Marginalized Versus General Populations

Several factors associated with Covid-19 VH among marginalized populations that were identified in this review are similar to those described among the general population, including vaccine-specific and individual-level concerns about Covid-19 vaccine efficacy, side effects, safety, perceived Covid-19 risk, and concerns about interactions with current medications among those with underlying medical conditions [27, 52]. However, while systematic reviews of Covid-19 VH among the general population in the U.S. [52] and Canada [27] identify significantly greater VH among Black and Latinx vs. White populations, race/ethnicity is predominantly addressed as an individual-level demographic factor; many studies do not include analyses of social and structural determinants of under-vaccination, including institutional racism and historically justified mistrust of government and public health authorities [40, 41]. As a result, racial/ethnic disparities identified in general population studies are often attributed to individual VH rather than critically assessed as a function of structural marginalization. Moreover, the lack of identifiable data on LGBTQ+ , American Indian/Indigenous, Asian/Asian-American, people with disabilities, and other marginalized demographics in general population samples—due to minimal subsample sizes and lack of specific demographic questions—precludes analyses of disaggregated data that would enable comparisons of factors associated with VH and under-vaccination among these populations versus the general population.

## Limitations and Strengths

Our findings should be understood in the context of study limitations. The overall objectives were to map and synthesize evidence on Covid-19 VH and under-vaccination among marginalized populations; we cannot draw causal conclusions about the associations identified, nor was our aim to quantify Covid-19 vaccination intentions or uptake, more apropos of meta-analysis. However, our scoping review methods reveal nuanced information through synthesis of quantitative and qualitative findings on shared and population-specific barriers to Covid-19 vaccination, and indicate directions for future research and interventions. Moreover, additional populations to those included might be characterized by marginalization, although we based our focal populations on previous evidence and a priori criteria [51, 53]. Additionally, certain population findings were based on scant empirical data, which may not represent the breadth and diversity within these populations. This indicates the need for further research on VH and under-vaccination among American Indians, Native Hawaiians, and Indigenous peoples, Asians/Asian Americans, Arabs/Arab Americans, persons experiencing homelessness, people who use drugs, and immigrants and refugees. Challenges also arose due to differences in the labelling of factors that influence VH and different emphases across studies. For example, medical mistrust was often labelled as such but variably attributed to structural racism and institutional stigma, conflated with trust in an individual HCP, or approached as an individual psychological characteristic. However, we held ongoing research team meetings to ensure consistent application of shared, a priori criteria in data extraction and categorization across the 4 domains, thus increasing methodological rigor. Finally, studies published after our review timeframe may represent additional populations, data, and study locales; however, we scoped over 10,000 sources and included over 100 peer-reviewed articles.

## Conclusion and Implications

In conclusion, we identified multilevel factors that influence Covid-19 VH and under-vaccination among marginalized populations, which may apply to other vaccines and future public health emergencies. This review suggests the importance of future research, using qualitative and quantitative methods, that distinguishes among vaccine-specific, individual, social/community, and structural factors associated with Covid-19 VH and under-vaccination. More specifically, it is crucial to identify and discern population- and culture-specific factors, as well as shared factors, that are associated with VH among marginalized communities—i.e., that fuel decisional ambivalence more appropriately defined as Covid-19 VH: “a state of indecision and uncertainly about vaccination before a decision is made to act (or not act)” [43,p.58]. This is foundational to the development and testing of evidence-based, culturally tailored interventions to reduce Covid-19 VH among marginalized populations, with methods that may be applicable to addressing VH in regard to other adult as well as childhood vaccines.

At the same time, this review suggests that it is vital that future research and interventions distinguish and address structural factors that undergird widespread under-vaccination among marginalized populations; this entails differentiating structural and institutional barriers in access—including those due to racism, homophobia/transphobia, ableism, xenophobia, and other forms of marginalization that are manifested in institutional policies and practices—from individual and social attitudes, beliefs, and misinformation that may underlie VH. Unilaterally referring to the array of multilevel factors identified in this review as vaccine hesitancy does a disservice to efforts to understand and intervene in under-vaccination during the Covid-19 pandemic. To this end, research, interventions, and policy measures to address Covid-19 VH and under-vaccination should meaningfully engage representatives of marginalized communities in problem identification, intervention design and implementation, and evaluation, as in the broader arena of pandemic preparedness [167, 172, 173]. Further, it is crucial to implement and assess the effectiveness of structural interventions in addressing under-vaccination. This includes population-specific public health initiatives to promote access to vaccination, such as culturally tailored messaging delivered by trusted messengers via social media, community outreach, and in healthcare settings; local pop-up clinics and clinic staff training [168, 172, 174]; resourcing and scaling up of community partnerships and local health infrastructures [30, 37, 167, 172, 174]; and institutional and policy reforms to eliminate stigma and discrimination in healthcare systems, which extend beyond the traditional remit of public health.

## Supplementary Information

Below is the link to the electronic supplementary material.

Supplementary file1 (DOCX 13 KB)

## Data Availability

All data are provided in the manuscript.
