# Supplementary material for: Covid-19 Vaccine Hesitancy and Under-Vaccination among Marginalized Populations in the United States and Canada: A Scoping Review
Source: J Racial Ethn Health Disparities. 2023 Dec 20;12(1):413–34. doi: 10.1007/s40615-023-01882-1 (PMC11746967; doi:10.1007/s40615-023-01882-1)
Supplement: Supplementary file 1 — Supplementary file1 (DOCX 13 KB) [file 40615_2023_1882_MOESM1_ESM.docx]

**Supplementary Table 1. Sample search string**

| **Medline and Embase** |
| --- |
| (((("corona virus" or coronavirus or COVID or nCoV) adj3 ("19" or "2019" or novel or new)) or "corona virus19" or "corona virus2019" or coronavirus19 or coronavirus2019 or COVID19 or COVID2019 or nCOV19 or nCOV2019 or "SARS Corona virus 2" or "SARS Coronavirus 2" or "SARS-COV-2" or "Severe Acute Respiratory Syndrome Corona virus 2" or "Severe Acute Respiratory Syndrome Coronavirus 2").ti,ab,hw,kw. and ((vaccin* or immunis* or immuniz*) adj3 (hesitanc* or mistrust or distrust or refus* or barrier* or confidence).ti,ab,hw,kw.)) |
